# Intracellular Regulation of Matrix Metalloproteinase-2 Activity: New Strategies in Treatment and Protection of Heart Subjected to Oxidative Stress

**DOI:** 10.1155/2013/130451

**Published:** 2013-12-24

**Authors:** Grzegorz Sawicki

**Affiliations:** ^1^Department of Pharmacology, College of Medicine, University of Saskatchewan, 107 Wiggins Road, Saskatoon, SK, Canada S7N 5E5; ^2^Department of Clinical Chemistry, Medical University of Wroclaw, Wrovasc Integrated Cardiovascular Centre, 50-556 Wroclaw, Poland

## Abstract

Much is known regarding cardiac energy metabolism in ischemia/reperfusion (I/R) injury. Under aerobic conditions, the heart prefers to metabolize fatty acids, which contribute to 60–80% of the required ATP. During ischemia, anaerobic glycolysis increases and becomes an important source of ATP for preservation of ion gradients. With reperfusion, fatty acid oxidation quickly recovers and again predominates as the major source of mitochondrial oxidative metabolism. Although a number of molecular mechanisms have been implicated in the development of I/R injury, their relative contributions remain to be determined. One such mechanism involves the proteolytic degradation of contractile proteins, such as troponin I (TnI), myosin heavy chain, titin, and the myosin light chains (MLC1 and MLC2) by matrix metalloproteinase-2 (MMP-2). However, very little is known about intracellular regulation of MMP-2 activity under physiological and pathological conditions. Greater understanding of the mechanisms that govern MMP-2 activity may lead to the development of new therapeutic strategies aimed at preservation of the contractile function of the heart subjected to myocardial infarction (MI) or I/R. This review discusses the intracellular mechanisms controlling MMP-2 activity and highlights a new intracellular therapeutic direction for the prevention and treatment of heart injury.

## 1. MMPs General Characteristics

Matrix metalloproteinases (MMPs) are proteolytic enzymes, which play significant roles in a range of physiological processes including morphogenesis, cartilage and bone repair, wound healing, cell migration, and angiogenesis. They are best known for their role in degradation of extracellular proteins and remodeling of the extracellular matrix. MMPs belong to a family of more than 25 enzymes that are not only structurally related but are also similar in substrate specificity [1–4]. Of these, the best known enzyme is matrix metalloproteinase-2 (MMP-2 or gelatinase A) and matrix metalloproteinase-9 (MMP-9 or gelatinase B). MMP-2, a constitutive enzyme, is found in almost all cell types and it degrades denatured collagen (gelatin) and collagen type IV (a component of the basement membrane) as well as other extracellular matrix proteins [5–7]. MMP-9 is a cytokine inducible MMP which is most commonly located in leukocytes [8–10]. Enzymes of this family possess a signal peptide, amino-terminal propeptide, a catalytic Zn binding site, and carboxy terminus domains [11–13].

Enhanced activity of MMPs, such as MMP-2 and MMP-9, is implicated in a variety of cardiovascular pathological states, including atherosclerosis, restenosis, ischemic heart disease, and heart failure [14, 15]. But attention was always given to the effects of MMP activities in tissues on a long-term scale (days or weeks) and to the associated changes in the extracellular matrix (protein degradation and remodeling of extracellular matrix). 

## 2. Regulation of MMP Activity

Similar to other proteolytic enzymes, all MMP activity is regulated at several levels such as transcription, regulation of mRNA half-life, secretion, intra- or extracellular localization, enzyme activation, and inhibition by specific and nonspecific proteinase inhibitors [16–25]. Described below are the three basic mechanisms: activation by proteolytic removal of propeptide, regulation of proteolytic activity by inhibitors, and chemical modification of enzyme molecule.

### 2.1. Regulation by Proteolytic Cleavage of Proenzyme

MMPs are expressed as latent enzymes. They can be activated by proteolytic cleavage of the N-terminal propeptide by a membrane-type MMP (MT-MMP) [18, 20, 22–24]. In this mode, the so-called “pro-MMP-2” (72 kDa) is activated by removing N-terminal part of proenzyme by action of a membrane-type MMP (MT-MMP) [18, 22, 24, 26] to its enzymatically active form, which is shorter by approximately 10 kDa (64 kDa). The proteolytic removal of the propeptide region perturbs the binding of a cysteine thiol residue with the active zinc ion (Zn^+2^) site. This process is instrumental for the expression of proteolytic activity [17, 27–29].

### 2.2. Regulation by Proteinase Inhibitors

Similar to other enzymes, MMPs are regulated by naturally occurring inhibitors called tissue inhibitors of metalloproteinases (TIMPs) [13, 15, 30, 31]. To date, 4 TIMPs have been described, TIMP-1, TIMP-2, TIMP-3, and TIMP-4. TIMPs are tight binding proteins and relatively small molecules, between 20 and 30 kDa. It is worth mentioning that peroxynitrite (ONOO^−^), a very potent reactive oxygen species, may also alter the structural and binding characteristics of TIMPs, resulting in their dissociation or lower affinity to MMPs [32]. The modification of TIMP may also lower affinity to pro-MMPs, resulting in the activation or impaired regulation of MMP activity [32].

In addition to natural protein inhibitors of MMPs, a broad range of synthetic compounds with anti-MMP activity are described. Synthetic inhibitors such as o-phenanthroline (Phen), hydroxamates, and the tetracycline-class of antibiotics, of which doxycycline (Doxy) is the most potent [33–37], share the common characteristic of chelating Zn^2+^. In studies which involve use of MMP inhibitors, Doxy is the reagent of choice. The effect of Doxy used as an MMP inhibitor in experimental and clinical studies is reviewed extensively by Castro and Tanus-Santos [38]. Unfortunately, despite the fact that many researchers claim these inhibitors as selective/specific inhibitors of MMPs, these compounds always affect the action of other proteolytic enzymes in addition to inhibiting MMPs [39–41].

### 2.3. Regulation by Posttranslational Modification of Enzyme Molecule

Regulation of metabolic enzymes or proteins involved in signal transduction by posttranslational modifications such as acetylation, nitrosylation, or phosphorylation in *in vivo* conditions is well documented [42–47]. However, almost nothing is known about chemical posttranslational modifications of MMPs *in vivo* and their effect on MMP activity. However, it has been shown under *in vitro* conditions that low concentrations of reactive oxygen species (ROS) increased MMP activity [48, 49], while higher concentrations decreased MMP-2 activity [50]. The role of ROS in oxidative stress pertaining heart injury will be discussed later.

In addition to the proteolytical cleavage, the disruption of the Cys-Zn^2+^ bond can be also induced by oxidizing agents like peroxynitrite (ONOO^−^). Maeda's group [48, 49] showed that low concentrations of ONOO^−^ activate pro-MMP without removal of the autoinhibitory propeptide domain. In fact, in the presence of normal intracellular levels of glutathione, ONOO^−^ causes the S-glutathiolation of a sequence within this autoinhibitory domain of MMPs [51]. Thus ONOO^−^ may alter the conformation of enzyme molecules, leading to their inactivation and resulting in increased MMP activity [32, 52]. The activation of MMP-2 in the presence of ONOO^−^ and glutathione (GSH) together is greater than with ONOO^−^ alone [51]. Recently we showed that in addition to ONOO^−^ dependent modifications, phosphorylation of MMP-2 decreased its *in vitro* activity [53]. Also it was shown that phosphorylation of a proform of MMP-2 determines the enzyme conformation and response to ONOO^−^ [54]. The intracellular activation of MMP-2 by ROS and its possible consequences in heart diseases are reviewed in [55].

Thus, there is a natural mistake in MMP nomenclature, as the term “pro-MMP” is generally used to describe the molecule with higher molecular weight as a “latent” enzyme, assuming that only the molecule with lower molecular weight is active.

As mentioned above, all our knowledge about the role of posttranslational modifications on MMP activity originated from *in vitro* studies. Our most recent studies, using a model of I/R injury, showed that the phosphorylation of MMP-2 molecule is important for its activity [53] and that inhibition of dephosphorylation of MMP-2 protects heart from contractile dysfunction caused by I/R [56].

## 3. Discovery of the Novel Action of MMP-2 

Fifteen years ago, work of Marek Radomski's group from Edmonton, Canada (currently Dublin, Ireland), on the mechanisms of the regulation of platelet function by nitric oxide (NO) showed a novel pathway of platelet aggregation mediated by MMP-2 [57–60]. He and his coworkers, for the first time, characterized the ultrastructural localization and cellular translocation of MMP-2 in the stimulated human platelet [59]. This was the first piece of evidence showing that MMP-2 acts not only in the extracellular matrix but also at the cellular level, with a time frame of action of minutes rather than hours or days. Soon after that, the mechanism of platelet dysfunction encountered during the use of extracorporeal membrane oxygenation (ECMO) was also investigated [57]. This study showed that metalloproteinase inhibitors might provide a means to intervene in the state of dysregulated platelet aggregation seen in ECMO, ultimately, leading to preservation of normal platelet homeostasis and minimization of the deleterious consequences of this life-saving therapy. This study provided the foundation for the development of a new approach in the study of MMP biology.

Shortly after these findings, several new actions of MMP-2, unrelated to the extracellular matrix, were revealed. These actions include the degradation of big endothelin [61], calcitonin gene-related peptide [62], and monocyte chemoattractant protein-3 [63]. MMP-2 was also shown to mediate neurotoxicity in HIV-infected macrophages by cleavage of the chemokine stromal cell-derived factor-1 [64].

In 1999 a sarcomeric association of MMP-2 was found in hearts of patients with dilated cardiomyopathy [65, 66]. Soon after that, it was shown that the action of MMP-2 included not only the intracellular degradation of troponin I (TnI) [67] and myosin light chain 1 (MLC1) [68], but also the degradation of poly (ADP-ribose) polymerase in the nucleus [69]. While working on apoptosis in cardiomyocytes, Menon and colleagues also observed an intracellular action of MMP-2 [70]. In addition, a new intracellular function of MMP-2 as an active negative regulator of mitochondrial function during superimposed oxidative stress was reported [71]. Novel intracellular functions were also found for MMP-1 and MMP-3 as mediators of apoptosis [72, 73] and for MMP-9 as a mitochondrial enzyme which, when activated, decreases myocyte contractility in hyperhomocysteinemia [74]. Indeed, several reports now indicate that MMP-2 does not solely degrade proteins of the extracellular matrix (reviewed in [75–77]). The most recent studies, which use cardiomyocytes subjected to oxidative stress, revealed the presence of a truncated isoform of MMP-2 in intracellular space, and this form of MMP-2 is responsible for initiation of innate immunity [78].

## 4. MMP-2 in Acute Heart Injury 

The detrimental roles of matrix metalloproteinases in cardiovascular disease states such as atherosclerosis and heart injury have been postulated [12, 79, 80]. Richard Schulz group from Edmonton, Canada, studied the role of MMP-2 with respect to oxidative stress in the heart triggered by ischemia/reperfusion (I/R) injury. He has shown that MMP-2 contributes to acute cardiac dysfunction and its inhibitors protect the heart from this injury [81]. Subsequently, he has shown that an intracellular contractile machinery regulatory protein, troponin I (TnI), is degraded by MMP-2 in I/R injury [67]. This was the first report showing intracellular function of an “extracellular enzyme.” Furthermore, an intracellular imbalance between an endogenous tissue inhibitor of metalloproteinases-4 (TIMP-4) and MMP-2 was shown in I/R injury [82]. These important discoveries provided the base for a new paradigm pertaining to the site of MMP-2 activity. Also, these discoveries started the momentum for the development of novel pharmacological strategy in the treatment of I/R injury.

It was suspected that TnI is not the only intracellular protein degraded by MMP-2 in I/R heart injury. In order to address this, a combined pharmacological and functional proteomics approach was used to analyze protein changes in I/R hearts. In this experimental setting we discovered that myosin light chain 1 (MLC1) is another target of MMP-2 [68]. Additionally, the most recent study from our laboratory has shown that MMP-2 is involved in the damage of endothelial integrity in I/R hearts [83]. This damage is associated with increased protein release into coronary effluent; however, the molecular mechanism of this phenomenon still has yet to be elucidated. In 2010, myocardial MLC2 [84] and titin [85] were identified as new MMP-2 substrates in oxidative stress.

The novel aspects of MMP-2's action in heart injury have been reviewed by Schulz [86], the role of MMPs in cardiac diseases has been reviewed by Spinale [87], and the intracellular actions of MMPs have been reviewed by Hockenbery [77].

## 5. Oxidative Stress

The following is a brief introduction in order to better understand the role of oxidative stress and, more specifically, the role of reactive oxygen species (ROS) in heart injury.

Oxidative stress is a common cause of heart injury and reflects an imbalance between ROS and ability of an organism to remove or inactivate the reactive entities or to repair the resulting tissue damage. ROS are toxic molecules formed during a variety of normal and pathological biochemical reactions. They are highly reactive and unstable. Furthermore, their reactions may lead to DNA damage, mitochondrial malfunction, cell membrane damage, and eventually cell death, a phenomenon referred to as “oxidative stress.” Oxidative stress results from an imbalance between the formation and the neutralization of prooxidants. Examples of ROS include hydrogen peroxide (H_2_O_2_), hydroxyl radical (^•^OH), nitric oxide (NO), singlet oxygen (^1^O_2_), superoxide anion (O_2_^−^), peroxyl radical (ROO^•^), and peroxynitrite (ONOO^−^). ONOO^−^ is a very potent entity, being ~1000× stronger as an oxidizing agent than H_2_O_2_. Markers of ONOO^−^ formation (such as nitrotyrosines or isoprostanes) can be found in many disease states including, but not limited to, brain injury [88–91], heart [92–94], lung [95, 96], liver disease [97, 98], preeclampsia [99, 100], and inflammation [96, 101–104], among others.

All cell types are capable of producing ROS, and evidence suggests that ROS produced by cardiovascular cells play important roles in the development and progression of heart injury and disease. Recent experimental studies suggest causational roles for increased ROS in the development of contractile dysfunction following myocardial infarction (MI) and pressure overload [105]. However, the precise contribution of ROS in cardiovascular pathologies remains unknown. The present review addresses this issue, specifically the action of ONOO^−^ on the molecular mechanisms of heart function. ONOO^−^ results from an extremely rapid reaction between ^•^NO and ^•^O_2_^−^. It is then protonated to form the chemically unstable intermediate, peroxynitrous acid (ONOOH), which rapidly decomposes to form highly reactive oxidant species including ^•^OH, nitrogen dioxide radicals (^•^NO_2_), and the nitronium cation (NO_2_^+^) [106].

The coupling between cardiac energy usage and contractility makes the heart a unique and excellent organ model for investigating the mechanisms of oxidative stress (e.g., triggered by ischemia/reperfusion (I/R) injury) and for determining the efficacy of new therapeutic agents. Despite the variations in the anatomy and physiology of different organs, the basic molecular and cellular mechanisms of all I/R injury and protection are essentially similar throughout the body.

## 6. Pathophysiological Roles of Reactive Oxygen Species in the Heart 

The general role of reactive oxygen species (ROS) in I/R injury has been extensively researched, with many studies showing a burst in ROS generation at the start of reperfusion (reviewed in [107]). However, increased ROS formation during ischemia remained unverified until our most recent work showed an increased production of ONOO^−^ during ischemia in isolated myocytes [108].

Whether or not NO plays a protective or detrimental role in I/R injury has been a controversial issue that requires an understanding of the biology of NO, O_2_^−^, and ONOO^−^ to resolve. The critical physiological balance between cellular concentrations of NO, O_2_^−^, and superoxide dismutase favours NO production, but under pathological conditions such as I/R or H-R, ONOO^−^ production is increased (reviewed in [109]). Within the normal heart, NO is synthesized by cardiac myocytes, endocardial endothelial cells, and vascular endothelial cells using NO synthase (NOS), known as endothelial NOS (eNOS) [110]. The production of NO is important for maintaining a variety of physiological functions within the heart such as setting coronary vasodilator tone, modulating myocardial contractile function, and providing an antioxidant environment [111]. The role of NO in delayed pharmacologically induced preconditioning was revived by Xi and Kukreja [112]. The cardioprotective effect of ONOO^−^ was observed for the first time by Lefer's group [113]. He and his colleagues showed that infusion of low concentrations of ONOO^−^ (2 *μ*M) protected heart from myocardial ischemia and reperfusion injury. Also, it has been shown that oxidant species, such as ^•^HO and ONOO^−^, trigger late preconditioning against myocardial stunning [114]. Very recently, Liaudet's group showed that ONOO^−^ is a key mediator for induction of cardioprotective mechanism during postconditioning [115]. On the other hand, Schulz et al. have also shown a cytotoxic effect of NO on heart tissue [116], and despite the evidence that ONOO^−^ contributes to I/R injury, its precise role remains unsolved [117–120]. A search for critical targets of ONOO^−^ or NO is a new approach to minimizing or preventing injury to the heart during oxidative stress. Work done by myself and others [48–50, 121], showing that ONOO^−^ activates matrix metalloproteinases (MMPs), and that MMPs have novel intracellular functions [57, 58, 60–65, 69],prompted the proposed investigation. 

It is significant to point out that ROS act on many targets, including kinases. For example, mitochondrial ROS initiate the phosphorylation of p38 MAP kinase [122], activate additional MAPK pathways [123] such as protein kinase B [124] and Src tyrosine kinases [125], and can induce nitration of PKCepsilon by NO facilitating its interaction with RACK2 for translocation and activation [126].

This review will help to clarify the role of ROS, including ONOO^−^ and phosphorylation, on potential MMP substrates and explain the mechanism of intracellular regulation of MMP-2 in physiology in hopes of better understanding its regulation in heart injury triggered by oxidative stress.

## 7. Intracellular Regulation of MMP-2 Activity

In general, MMP production and activity *in vivo *are strictly regulated. What is new, and to my knowledge, not yet broadly known and investigated in relation to oxidative stress, is the regulation of proteolytic activity of proteinases (not only MMPs) by the posttranslational modifications that render normally proteolysis-resistant proteins susceptible to these enzymes. The discovery of new sarcomeric substrates for MMP-2 in injured heart, such as TnI, titin, MLC1, and MLC2, raised questions concerning the intracellular regulation of MMP-2.

Most studies in this area were done with the use of MLC1 and MLC2. MLCs were used to address these questions because they already were known to be key proteins affected by oxidative stress [68, 84], and their chemical properties favoured them over other contractile proteins. The other contractile proteins are very difficult to study when techniques involved in studying protein electromobility are used. TnI is a very basic protein (pI ~ 10.5) and therefore is very difficult to use in studies involving 2-dimensional electrophoresis. Titin is a huge protein (~3.5 MDa) and is also very difficult to study with techniques involving electrophoretic separation.

Most recently, we have shown the involvement of ONOO^−^ in nitration and nitrosylation of MLC1 and MLC2 from hearts subjected to H-R, and that these modifications increased their degradation by MMP-2 [84, 127]. These observations were confirmed in another model of oxidative stress, isolated cardiomyocytes subjected to hypoxia-reoxygenation (H-R), in which the ONOO^−^ scavenger FeTPPS protected against H-R-induced contractile dysfunction [108]. We have shown that *in vitro,* phosphorylation and nitration of MLC1 increase its degradation by MMP-2 [108]. Also, our most recent data from animal model of I/R injury [128] and H-R with use isolated cardiomyocytes [108] show phosphorylation, nitration, and nitrosylation of MLC1 from I/R hearts. Phosphorylation and nitration of the crucial residues were found only in the MLC1 from I/R hearts; therefore, these modifications may be responsible for sensitizing MLC1 to degradation by MMP-2 and thus may play an important role in regulating the intracellular action of MMP-2 during I/R injury. Collectively, our data, together with other reports [129–132], suggest that studying MLC posttranslational modifications could provide new information that will lead to the discovery of the missing link in the mechanism of intracellular regulation of MMP-2 activity. These discoveries would provide support for a new paradigm pertaining to the regulation of MMP-2 activity and to the site of MMP-2 action and the impetus for the development of a novel pharmacological strategy in the treatment of oxidative stress (I/R or H-R) injury.

To summarize, the detrimental role of ROS and increased MMP-2 activity on heart function is documented. However, the myocardial protein targets for ROS are still not fully identified and the molecular consequences of ROS action remain unknown. This is why my general hypothesis is that the oxidative stress triggers posttranslational modifications of contractile proteins and most likely other proteins as well. Following these changes, the proteins become substrate for MMPs. Oxidative stress also increases MMP activity, which was evidenced by us and others [133–136], and this increased activity contributes to the impairment of myocardial contractile function by degradation of previously proteolysis-resistant contractile proteins. Thus, the pharmacological inhibition of contractile protein modification, together with the inhibition of MMP activity, should provide adequate protection to the heart from MI, I/R, or generally speaking, from injury triggered by oxidative stress.

## 8. Modifications of MLCs Triggered by Oxidative Stress in Isolated Rat Heart

Very little is known about the regulation of MMP-2 activity in the myocardium. While we have already shown that the balance between MMP-2 and TIMP-4 is disrupted in the heart subjected to I/R injury [82], there must also be other intracellular mechanisms of MMP-2 regulation.

Using our heart contractility recovery criteria [137] and 2-dimensional electrophoresis, we found the level of the truncated form of MLC1 in I/R hearts to be approximately double that in aerobic controls [128]. This increased level of the truncated form of MLC1 was negatively correlated with recovery and positively correlated with increased MMP-2 activity, which was correlated with the duration of ischemia [137]. However, while immunoblotting analysis showed a 40% decrease in total MLC1 in the I/R group (data not published), the level of intact MLC1 (>95% content of total MLC1), as measured by 2-dimensional electrophoresis, remained unchanged (data not published). This discrepancy could be explained by decreased immunoreactivity of MLC1 caused by posttranslational modifications inside an epitope of MLC1, triggered by I/R injury. This phenomenon is not unique; similar discrepancies have been reported in actin levels in an *in vivo* model of MI [138] and in an islet amyloid peptide epitope from diabetic patients [139].

Our mass spectrometry analysis showed qualitative and quantitative differences between MLC1 from control and I/R hearts (data not published). Data from the analysis of peptide mass fingerprints (PMF) showed similar amounts of modified and nonmodified peptides of intact MLC1 isolated from aerobic and I/R hearts, but twice as many peptides with posttranslational modifications in the truncated form of MLC1 from I/R hearts. In summary, oxidative stress triggered by I/R caused extensive posttranslational modifications of the MLC1 molecule. We also have substantial preliminary evidence demonstrating that MLC1 from I/R is phosphorylated [128], nitrated, and nitrosylated [108]. *In vitro *experiments showed enhanced degradation of phosphorylated MLC1 and that phosphorylated MLC1 had a higher affinity for MMP-2 than the nonphosphorylated form [128]. Similarly, nitration/nitrosylation of MLC1 caused by ONOO^−^ treatment increased MLC1 degradation, with a correlation between the degree of degradation and ONOO^−^ concentration [108]. While nitration of tyrosine is considered as a key target of ONOO^−^ action, phenylalanine nitration by ONOO^−^ is also documented [140–142] although very little is presently known about it.

## 9. Identification of the Chemical Modifications of MLCs and MMP-2 Triggered by Oxidative Stress and Their Role in Isolated Cardiac Myocytes

Our work and that of others have shown that (a) oxidative stress injury caused by I/R [67, 68, 81, 82], infusion of ONOO^−^ [143] or cytokines [144] into the myocardium, or H-R [145] increases the activity of MMP-2; (b) this activity is positively correlated with the degree of injury [79, 137]; and (c) MMP-2 is colocalized within the sarcomeres of cardiac myocytes with TnI, titin, MLC1, and MLC2 [67, 68, 84, 85]. All of these observations, made in intact, isolated hearts and in the rapid time course of the injury process (minutes), appear to be independent of changes in collagen content [82, 144]. For the reason that the heart is a very complex organ, it is important to identify the precise location of MMP-2 action. To confirm that our isolated heart model findings reflect the intracellular actions of MMP-2, use of isolated myocytes subjected to H-R is fully justified. This biological model indicates whether the intracellular action of ROS in a single cell directly modulates sarcomeric MLC levels and/or triggers chemical modifications and if these modifications are associated with intracellular MMP-2 action on sarcomeric MLC1 and MLC2. Modifications of intracellular MMP-2 and changes in its activity can be investigated as well. 

Using isolated cardiac myocytes, it has been shown that ONOO^−^ caused a concentration-dependent reduction of myocyte contractility. This increased MMP-2 activity and the infusion of MMP inhibitors, doxycycline or PD 166793, attenuated the reduced contraction induced by ONOO^−^ [146]. This was the first demonstration that inhibition of MMPs could protect the isolated cardiac myocyte from ONOO^−^-induced contractile failure, and that MMPs cause this via a mechanism of action unrelated to proteolysis of extracellular matrix proteins. In our most recent work [108] using isolated cardiomyocytes subjected to 60 minutes of simulated ischemia, ONOO^−^-modified MLC1 increased degradation of MLC1 by MMP-2, and ONOO^−^ scavengers protected cardiomyocytes from contractile dysfunction triggered by oxidative stress.

My group showed that MLC1 from isolated myocytes subjected to simulated ischemia is S-nitrosylated and nitrated. These data show that the posttranslational modifications of MLC1 are most associated with nitration of tyrosine 190 and tyrosine 78 and S-nitrosylation of cysteine 81. For the reason that the observed posttranslational modifications in MLC1 from myocytes subjected to simulated ischemia are similar, but not identical to those observed in MLC1 isolated from I/R hearts, use of the chemically induced H-R model [147–149] is a better choice than the simulated ischemia model.

## 10. Protection of the Heart from H-R-Induced Contractile Dysfunction in a Model of Neonatal Asphyxia

It is important to understand whether the observations involving modifications of MLCs in I/R and H-R injury are a common phenomenon related to the nature of oxidative stress. To address this question a piglet model of neonatal asphyxia (H-R model) is a good choice. Neonatal asphyxia is a clinical condition that affects millions of newborns worldwide annually. Using a piglet model of neonatal asphyxia, decreased levels of MLC1 and MLC2 found were in the heart of animals subjected to H-R, which were associated with an increased level of MMP-2 [84, 127]. In contrast to I/R studies, only ONOO^−^-dependent modifications of MLCs were observed in this model of H-R [84, 127].

## 11. Final Remarks and Possible Clinical Implications

Phosphorylation of MLC1 has been previously described [129, 150]. While speculation involving its physiological role is still related to the modulation of contractile force, the pathological implications of MLC1 phosphorylation are not understood. Degradation of MLC1 during oxidative stress and I/R injury has been postulated and established [68, 108, 127, 151]. Nonetheless, the mechanism by which proteolytic degradation of contractile proteins occurs remains to be established. Phosphorylation of MLC1 has been shown in physiological conditions [151] as well as in chemically induced preconditioning [129]. However, the mechanism of MLC1 phosphorylation and its role in cardiac function are far from being understood.

In this review I focused on the intracellular mechanism by which MMP-2 acts on MLCs, but mostly on oxidative stress-mediated intracellular modification of MLC1 through phosphorylation, nitration or nitrosylation. This modification results in an increased degradation of MLC1 and MLC2 by the proteolytic enzyme MMP-2 that results in decreased mechanical function of the injured heart. Furthermore, I discussed the possible prevention of both the degradation of MLC1 and reduction in contractile function of the I/R heart by both inhibition of MLC phosphorylation and MMP-2 activity. I have also discussed the novel possible pharmacological approach when inhibitors of MLC modifications are infused to the I/R heart simultaneously with inhibitors of MMP-2 activity. In such a therapeutic scenario a synergistic protective effect is observed in regard to the contractility of injured hearts. 

The concept of reperfusion injury is recognized as a clinical phenomenon that may result in microvascular damage or myocardial stunning. The final consequence of this event is left ventricular systolic dysfunction leading to increased morbidity and mortality. The typical clinical case of reperfusion injury occurs in acute MI in which an occlusion of a coronary artery is followed by reopening of the artery. Another case is coronary artery bypass graft surgery (CABG).

Currently, the most important goal of pharmacological prevention and therapy in the course of heart disease, including MI, is to improve the oxygen supply/demand ratio for the heart [152–154]. For the prevention and treatment of MI, current clinical practice involves a multidrug regimen targeting restoration of normal coronary blood flow and/or decrease of myocardial oxygen consumption. Agents which either clinically or experimentally reduce the extent of myocardial injury when administrated just prior to reperfusion involve cell membrane receptor agonist adenosine, angiotensin-converting enzyme inhibitors, opioids and erythropoietin or the mixed cell membrane, and intracellular agonists [155]. Currently, more than 90% of patients on hospital arrival with suspicion of acute MI are given aspirin and/or a *β*-blocker [156]. Further treatment is based on analysis of factors including beginning and duration of symptoms, patient history, electrocardiogram, and physical examination of the patient.

Because there is no agent universally accepted to prevent or treat reperfusion injury, new directions in pharmacological treatment of the injured heart or protection from MI are needed and are under investigation. Therefore, it is important to unravel mechanisms involved in heart injury and to develop new pharmacological strategies for the treatment of MI.

On the basis of the information provided here I am almost positive that in addition to the existing approaches in clinical practice for protection and treatment of heart injury from oxidative stress, MMP inhibitors and inhibitors of modifications, like nitration, hydroxylation, S-nitrosylation, and phosphorylation, hold great potential as novel strategies to reduce the impact of ischemic heart disease. Recent studies by Ceriasano's group showed that doxycycline (inhibitor of MMPs) therapy in patients with acute MI and left ventricular dysfunction prevents the progression of adverse remodeling myocardium [157]. The studies presented in this review show that testing potential therapeutic agents for synergism in reperfusion protection should lead to the development of a “drug cocktail” or a “super pill” to protect the hearts of human patients from I/R injury. The concept of protecting I/R hearts using the synergistic action of drugs inhibiting modifications of contractile proteins together with inhibition of MMP-2 activity is currently under investigation in my lab and has been reviewed [158, 159].

Because the basic molecular and cellular mechanisms of all I/R injury and protection are essentially similar throughout the body, this concept is important to all pathologies involving I/R, including transplantation or reattachment of limbs. Finally, this novel approach will provide evidence to support the development of new directions in the study of the mechanisms of heart failure.
